# Lasered Graphene Microheaters Modified with Phase-Change Composites: New Approach to Smart Patch Drug Delivery

**DOI:** 10.3390/mi13071132

**Published:** 2022-07-18

**Authors:** Victoria Gilpin, Deetchaya Surandhiran, Cameron Scott, Amy Devine, Jill H. Cundell, Chris I. R. Gill, L. Kirsty Pourshahidi, James Davis

**Affiliations:** 1 School of Engineering, Ulster University, Jordanstown BT37 0QB, Northern Ireland, UK; gilpin-v@ulster.ac.uk (V.G.); deetchaya_s-ds@ulster.ac.uk (D.S.); scott-c55@ulster.ac.uk (C.S.); devine-a14@ulster.ac.uk (A.D.); 2 School of Health Sciences, Ulster University, Jordanstown BT37 0QB, Northern Ireland, UK; jh.cundell@ulster.ac.uk; 3 School of Biomolecular Sciences, Ulster University, Coleraine BT52 1SA, Northern Ireland, UK; c.gill@ulster.ac.uk (C.I.R.G.); k.pourshahidi@ulster.ac.uk (L.K.P.)

**Keywords:** curcumin, phase-change material, drug delivery, thermal activation, smart patch, laser-induced graphene (LIG)

## Abstract

The combination of paraffin wax and *O*,*O*′-bis(2-aminopropyl) polypropylene glycol–*block*–polyethylene glycol–*block*–polypropylene glycol was used as a phase-change material (PCM) for the controlled delivery of curcumin. The PCM was combined with a graphene-based heater derived from the laser scribing of polyimide film. This assembly provides a new approach to a smart patch through which release can be electronically controlled, allowing repetitive dosing. Rather than relying on passive diffusion, delivery is induced and terminated through the controlled heating of the PCM with transfer only occurring when the PCM transitions from solid to liquid. The material properties of the device and release characteristics of the strategy under repetitive dosing are critically assessed. The delivery yield of curcumin was found to be 3.5 µg (4.5 µg/cm^2^) per 3 min thermal cycle.

## 1. Introduction

The advantages of transdermal drug delivery are well established and can offer a simple, painless method of administering therapeutics without the issues associated with oral ingestion or subcutaneous injections [1,2,3]. The avoidance of first-pass metabolism also provides an opportunity to lower the dosage and minimise potential side-effects. The efficacy of transdermal patches can be evidenced through the availability of numerous commercial products that include nicotine [4,5], estradiol [6], fentanyl [7,8], rivastigmine [9], rotigotine [10], and diclofenac [11,12]. While the commercialisation of transdermal patches has been ongoing since 1979, it must be noted that, in the majority of cases, delivery is predominantly via passive diffusion with the rate of transfer to the patient dependent upon the formulation of the patch itself. Transfer is initiated once the patch is applied and continues until the drug reservoir is exhausted; as such, there is no additional/external means of adjusting the dosing regimen beyond the physical removal of the patch itself. In this communication, a novel release strategy based on the thermally controlled transfer of a model drug is considered with a view to enabling repetitive transdermal dosing.

Transdermal patches and microdevices that responsively alter a drug’s release dynamics upon being activated by mechanical, thermal, light, or electrical activation have begun to emerge in recent years [13,14,15,16]. In this particular case, the electrothermal activation of a phase-change material (PCM) was explored as a means of enabling the real-time modification of dosage. The strategy is based on the preparation of a composite comprising an alkane blend incorporating a modified amino-functionalised polyethylene glycol polymer within which the model drug is dispersed. The use of paraffin wax as the principal PCM component was chosen on the basis of its inherent biocompatibility but also the fact that the formulation can be manipulated to cover a variety of temperature transitions (the commercial variant used here melts at 43 °C); thus, the patch can be tuned to particular applications. The delivery mechanism relies on the transition of the PCM film from solid to liquid upon reaching a temperature of 43 °C (low enough to avoid pain/damage to the skin but high enough to minimise exogenous activation).

The innovative design approach adopted here involves combining the film of the PCM–drug composite with an on-patch microheater element based on a layer of laser-induced graphene (LIG). Interest in the use of LIG-based devices have increased tremendously in recent years with the ability to rapidly prototype devices through the additive manufacturing processes—principally scribing of suitable polymeric films (typically polyimide). The production of mechanically flexible films with tuneable conductivity and versatile interfacial chemistry can be ideally suited to the development of wearable sensing systems. It can also, as proposed here, be used for the actuation part, which opens up the possibility of closed-loop drug delivery devices [17,18,19,20].

**Figure 1:**
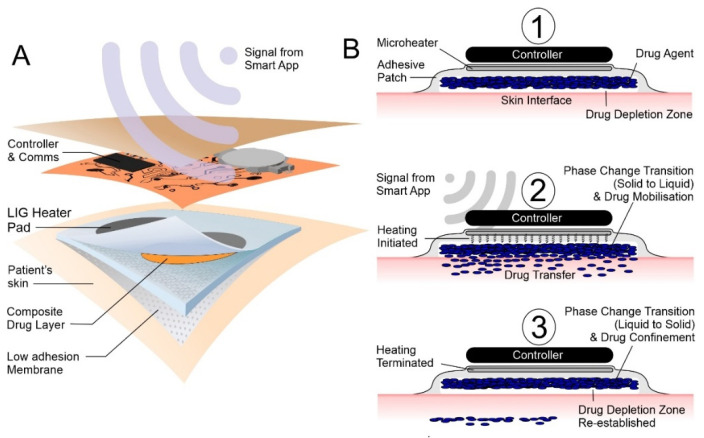
(**A**) Before activation, the drug is immobile and not in contact with the skin. (**B**) Activation initiates the heating cycle (1) facilitating phase change and mobilisation of the drug to the skin interface (2). Terminating the heating cycle (3), the system cools, phase change reoccurs, and the drug is once more rendered immobile.

At room temperature, the film containing the target drug (the therapeutic reservoir) is solid, which essentially renders the entrapped drug immobile. However, once the patch receives an appropriate signal, the graphene microheater increases the film temperature to 43 °C, inducing phase transition, mobilising the entrapped drug, and facilitating diffusion to the patch–skin interface (Figure 1B (1→2)). A critical advance here is that the drug would be expected to transfer only when the film is in the molten phase and the interface is being repeatedly replenished. The initial signal sent to the heater would also control the heat duration and, upon termination, the film would resolidify, and drug transfer would cease (Figure 1B (3)). Any drug remaining at the interface would be expected to transfer until phase transition whereupon the interface would be depleted (the majority of the drug would still remain within the internal bulk of the film). Recommencing the heating process remobilises the entrapped drug, effectively recharging the interface and delivering a second dose, thereby regulating repetitive dosing. In contrast to passive patches, it is conceivable that this approach would enable the complex delivery profiles for transdermal administration to be enacted.

Two agents were selected for investigation as model drugs: chlorophenol red (CPR) and curcumin. The former was chosen primarily as an analytical marker with the ease with which it can be detected, providing a facile route through which the operating characteristics of the proposed smart patch system could be readily determined. Curcumin, in contrast, is recognised as a versatile therapeutic agent possessing antimicrobial and anti-inflammatory properties, and there is an extensive literature base dedicated to its clinical application [21,22,23,24,25,26]. Curcumin has been found to be particularly valuable in wound healing, where it has been demonstrated to accelerate wound closure. As such, there have been considerable efforts to develop functional materials that can provide a mechanism for its controlled release. This has included complexation with cyclodextrin [21] or encapsulation within various polymer films [21,22], hydrogels [23,24], and nanoparticles [25], with most still relying on passive release once in contact with the wound fluid [26]. More recently, there have been reports of stimulus-responsive release (typically pH) [13,14,15,16], but no approach exists that can precisely modulate the release (i.e., scheduled delivery). The ability to control the delivery of curcumin through closed-loop sensing/actuation could offer a much more refined option for wound management (i.e., enabling maintenance of optimal dosage). The investigation reported here sought to assess the suitability of the proposed methodology as a foundation for both controlled delivery, whether for transdermal or wound care.

## 2. Materials and Methods

All chemicals were obtained from Sigma (Gillingham, Dorset, UK), were of analytical grade, and were used without any further purification. Paraffin wax pellets (76242) and *O*,*O*′-bis(2-aminopropyl) polypropylene glycol–*block*–polyethylene glycol–*block*–polypropylene glycol (AP-PEG) were used as phase-change materials to encapsulate the model drugs (chlorophenol red and curcumin). Britton Robinson (BR) buffer solutions were used throughout and were composed of equimolar acetic, boric, and phosphoric acids (each 0.04 M) adjusted to the required pH through the addition of sodium hydroxide. Raman spectroscopy was conducted using a Renishaw Raman Microscope (20× objective lens) with a 532 nm laser operating at 10% power. Conductivity measurements of the lasered polyimide were acquired with an Ossila Four Point Probe. A Thermo Scientific Genesys 150 dual-beam spectrometer (2 nm bandwidth) was used to acquire UV/Vis spectra. Disposable polymethyl methacrylate (PMMA) cuvettes (3 mL volume, 10 mm path length) were used throughout.

### 2.1. Lasered Graphene Heater Fabrication

**Figure 2:**
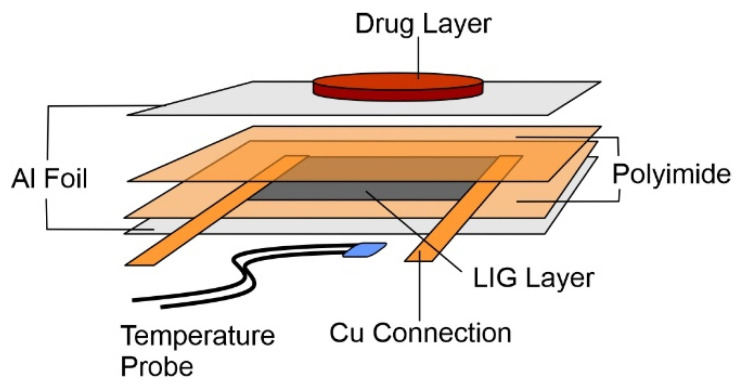
Schematic of the laser-induced graphene (LIG) heater components and their assembly.

The microheater was produced through the controlled laser ablation of polyimide film (127 µm thick, Dupont Kapton ^®^ 4-100-KHN-5, TapeCase, IL, USA) using a computer-controlled Atomstack A5 Laser, 5W Laser Diode, (445 nm). In the design employed (Figure 2), the LIG occupied a 15 mm by 15 mm square. The LIG layer scribed onto polyimide was measured by DekTak profiling before and after sonication, with the latter used to remove the carbonised layer such that the difference would provide the thickness of the LIG component. This enabled estimation of the cross-section area and conductivity (588.40 S/m). Adhesive copper tape was applied to two opposing sides of the LIG square to make electrical contact. A layer of Kapton polyimide tape was then placed over the top surface of the exposed LIG surface to provide some thermal insulation and help stabilise the temperature of the heater. A thin layer of aluminium tape was then applied to both the top and the bottom surfaces of the heater to act as a heat spreader, helping to dissipate the thermal energy more uniformly over the surface. The DS18B20 temperature sensor was then attached to the top side of the heater using a thermal adhesive, on the opposite side from the wax. This temperature sensor was used to monitor the current temperature of the heater, and it fed live data back to the control circuit. This allowed the control system to constantly monitor the temperature and turn the power going to the heater on or off in order to keep the temperature at the target level.

### 2.2. Heater Control

**Figure 3:**
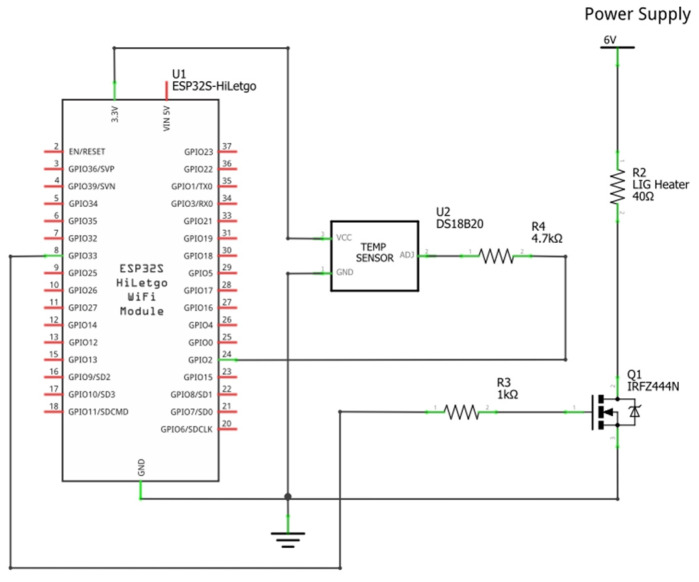
Schematic of the circuit design employed for the control of the LIG-based heater.

The electronic circuit (Figure 3) comprises an ESP32 microcontroller for control, a DS18B20 temperature sensor to monitor the heater, and a IRFZ44N N-channel MOSFET to act as a switch so that the power to the heater can be modulated. The MOSFET is placed on the low side of the heater, making/breaking the electrical connection between the heater and ground. Power to the heater is provided directly using a DC power supply (TENMA 72-10480, Farnell, City, Leeds, UK). Both the serial communication and the power for the ESP32 are provided using a micro-USB cable, connected to a computer. The DS18B20 sensor was selected due to its small formfactor and ability to daisy-chain multiple sensors together using the same wiring for one—something that could be useful for future development of a multichannel heating element controlling the delivery of many different drugs. 

Early into the development of the control circuit, the ESP32 was established as the microcontroller unit(MCU) to be used due to several key advantages: low cost, SPI/I2C communication support, and local availability. Future development beyond the current project scope is still possible using this MCU since it also can be surface-mounted via soldering to a custom printed circuit board (PCB), with support for Wi-Fi, Bluetooth, and Bluetooth Low Energy. This would enable the system to communicate with a web server or mobile phone app through the addition of a software update, without any modification to the hardware. 

A potential of 6 V was applied to the heater, drawing a current of 120 mA and dissipating approximately 0.72 W of power as heat. The time to target temperature (55 °C) was typically 2 min, although it should be noted that this time could be attenuated to shorter rise times by increasing the supply voltage. The 6 V/120 mA configuration was used throughout for convenience, and, while the system was powered from a bench-top supply, it was envisaged that this could be replaced by the use of a single-cell Li-ion battery (i.e., LP402025-IS-3, BAK). For example, on the basis of a total “on” time of 5 min targeting 55 °C, it could be expected that the circuit could be adapted to exploit a standard 3.7 V (165 mAh) cell and provide 9–10 thermal cycles before recharging.

### 2.3. Phase-Change Material—Drug Composite Films

Chlorophenol red (CPR) was used as the model drug in the early stages of development as it possesses water solubility and a spectroscopic signature which allows unambiguous (λ_max_ = 582 nm) and sensitive detection (ε = 19,449 L^−1^·mol·cm^−1^) using conventional UV/Vis spectroscopy (Section 2.4). The CPR component (typically 5% *w*/*w* loading) was mixed directly with molten paraffin wax (the phase-change material) and, once homogeneous, was cast into circular holders (1 mm thick, 10 mm diameter) and left to cool. The total mass of the resulting drug–wax composite was 90 mg. Prior to commencing the drug release tests, each disc was washed with copious amounts of deionised water in order to remove any CPR present at the wax interface. The hydrophobic nature of the wax should minimise penetration of the water; thus, the CPR encapsulated within the bulk should be retained.

A similar approach was initially used for those studies involving curcumin (typically 1% *w*/*w*), but a second formulation involving premixing with *O*,*O*′-bis(2-aminopropyl) polypropylene glycol–*block*–polyethylene glycol–*block*–polypropylene glycol (AP-PEG) was also utilised. In such instances, the solid curcumin was added directly with molten AP-PEG, and then resulting mixture added to molten wax to provide a wax/AP-PEG/curcumin formulation in the ratio 85:14:1 by weight.

### 2.4. Drug Release Studies

**Figure 4:**
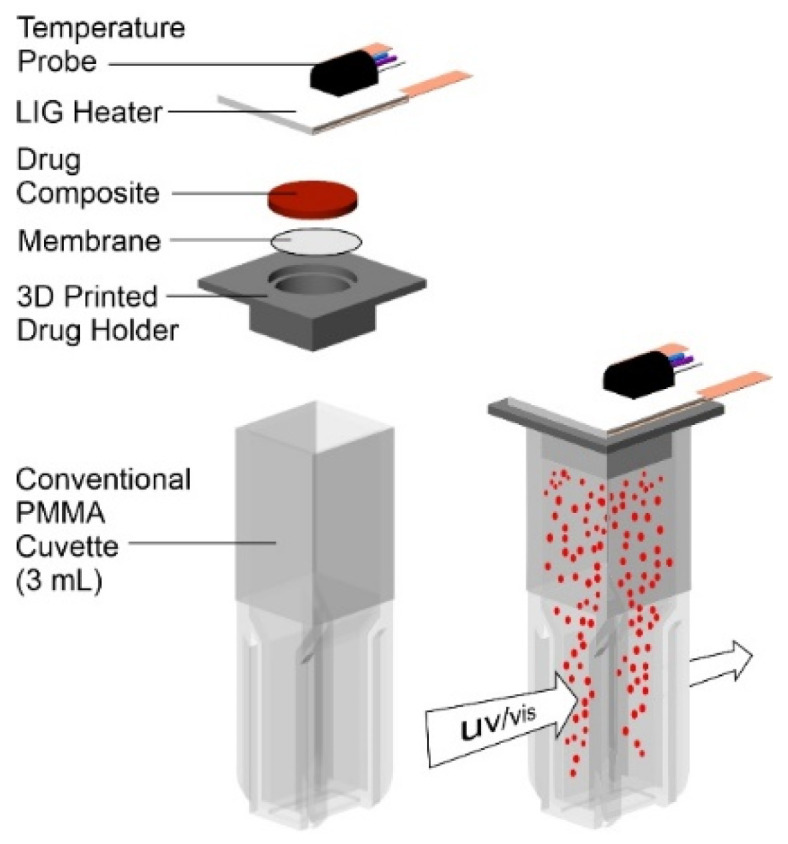
Spectroscopic cell configuration used to monitor the release of the model drug components as a consequence of heat applied from the LIG patch.

An in situ release measurement system was developed in order to accurately quantify the amount of drug being released without perturbing the actual patch assembly. This is a significant consideration where repeated dosing from the same drug reservoir can be conducted. Rather than using a conventional Franz cell with sampling arm, the approach adopted here was to locate the patch (drug film, heater, and associated electronics) onto a conventional cuvette. The configuration adopted is highlighted in Figure 4 and consists of a disposable 3D-printed flange which can be inserted into the top of the cuvette and contains a recess which allows placement of the drug composite disc with the heater situated flush on the extended flange component. The cuvette is filled with the appropriate receiving solution (typically pH 6 buffer). The drug–composite disc is separated from the receiving solution by a Durapore^®^ PVDF (0.1 micron) filter, which provides a degree of structural support to the wax composite whilst also aiding solution distribution across the interface. The molten wax is immiscible with the aqueous receiving solution, and, while it is possible that it could penetrate within the filter, it would nevertheless remain at the solution interface. As the filter is spatially distant from the UV/Vis beam, there should be no direct interfere with the measurement. Rather than removing aliquots of the solution, the cuvette can be placed directly within the spectrometer, and the absorbance of the receiving solution can be interrogated without incurring any errors associated with solution manipulation. Knowledge of the molar absorptivity for the particular drug being investigated can then enable the amount of drug being released to be quantified.

## 3. Results

### 3.1. Laser-Induced Graphene (LIG) Heater Characterisation

**Figure 5:**
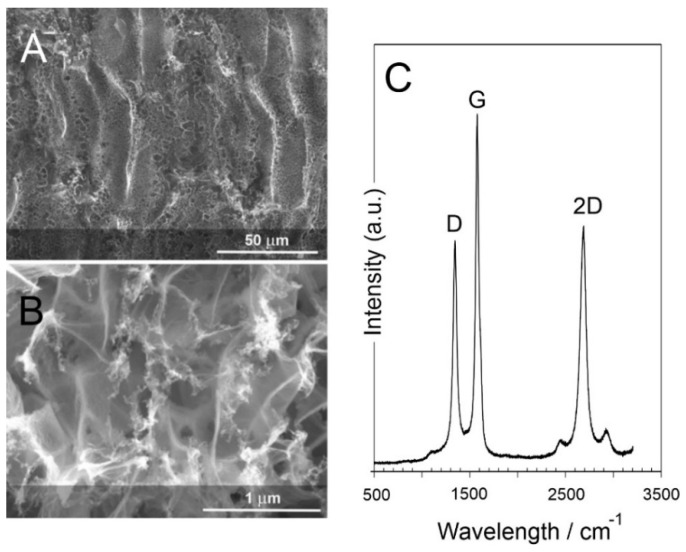
Scanning electron micrograph of the LIG film highlighting the micro/nano porosity (**A**) and abundance of fragmented structures (**B**). (**C**) Raman spectrum of the LIG film.

Scanning electron micrographs of the laser scribed polyimide film are detailed in Figure 5A,B. While the polyimide film is essentially featureless, the laser processing results in thermal degradation of the substrate with the release of gas inducing a foaming effect, which, upon cooling, leads to a raised carbon track. The successive rastering of the laser across the surface of the polyimide film leads to furrow-like patterning with micro/nano porosity clearly evident (Figure 5A). The general morphology of the resulting LIG film is, however, consistent with previous reports [17,20]. Raman spectroscopy was conducted to examine the nature of the carbon layer resulting from the laser processing, and a representative spectrum is detailed in Figure 5C. The D (1350 cm^−1^), G (1585 cm^−1^), and 2D (2700 cm^−1^) peaks common to carbon structures are clearly observed. While the G band is normally associated with graphitic in-plane vibration with an E2g symmetry, the D band is generally attributed to the presence of defects within the lattice and is an indicator of disorder [27,28,29]. The prominence of the latter is common with LIG-based materials, and it was further corroborated here through inspection of the highly fragmented structure observed in the electron micrograph highlighted in detail in Figure 5B.

The LIG heater was programmed to cycle to a target temperature of 55 °C. This was required in order to induce the complete phase transition of the wax–drug composite and, thus, enable the mobilisation of the entrapped drug and its release at the wax–solution interface. 

**Figure 6:**
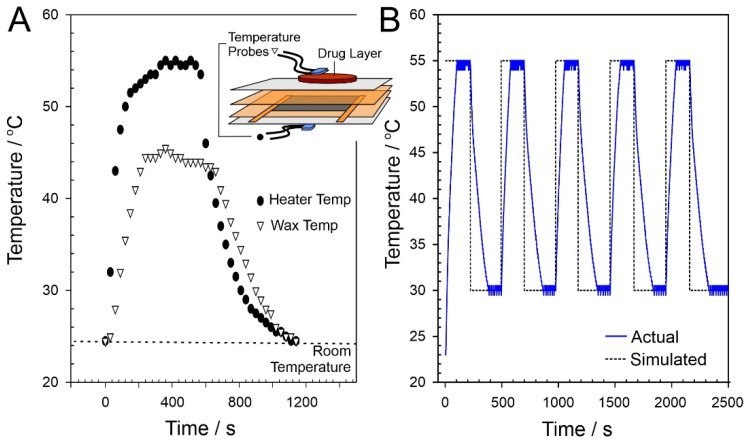
(**A**) Variation in measured temperature at the heater (circles) and drug–solution (triangles) interfaces. Inset: experimental configuration used to acquire temperature data. (**B**) Comparison of simulated and actual temperature profile at the LIG heater upon repetitive cycling.

A temperature gradient exists between the LIG heater and the wax surface which would be in contact with the receiving solution (and ultimately the skin of the wearer). As such, the heater must be at a higher temperature to drive the phase transition and ensure that drug transfer can occur across the entire interface. The methodology assumes that, initially, the interface is devoid of drug (as per the prewashing step) and that the interface becomes charged with drug only when molten. Incomplete transition to the molten phase could result in islands of solid wax which would reduce the effective surface area through which drug transfer can occur. The discrepancy in temperatures between the heating pad and the wax–solution interface is highlighted in Figure 6A. The activation temperature of 55 °C results in an interfacial temperature of 43 °C, sufficient for complete phase transition and drug transfer while minimising discomfort to the potential wearer.

**Figure 7:**
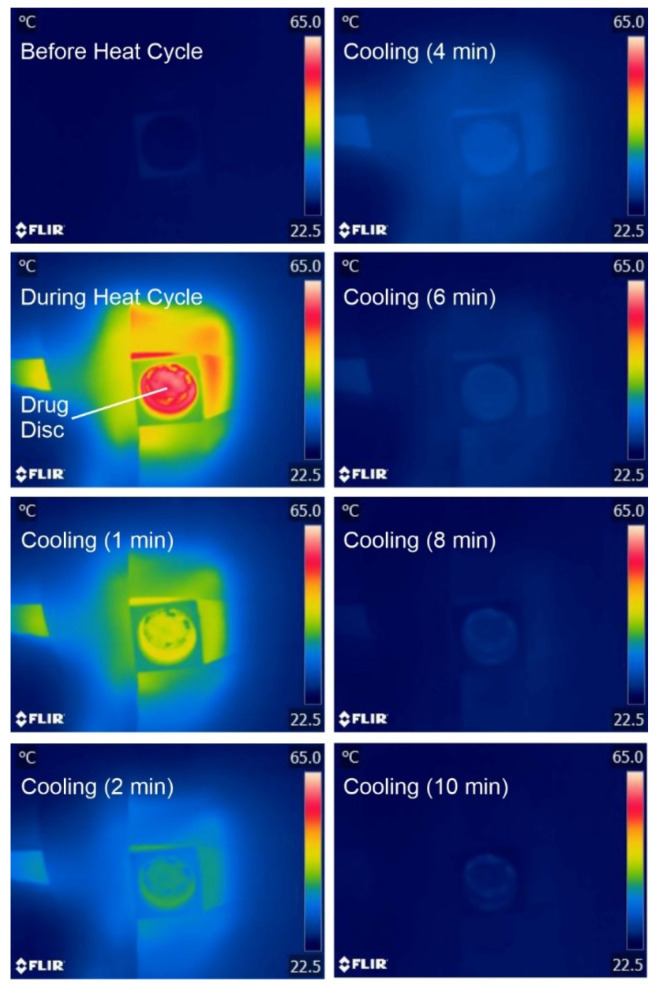
Thermal imaging of the drug film layer before, during, and after onset of heating at the LIG patch assembly.

The robustness of the LIG heater was assessed through performing repetitive cycling between 55 °C and 30 °C (nominal “off”). The skin temperature would typically be lower than the core temperature and can vary depending on the location of the body which is being sampled. Lee and co-workers (2019) found that the regional surface skin temperature (SST) could vary between 29.8 °C (sole of foot) and 35 °C (anterior of neck) [30]. The LIG heater was programmed to cycle between 30 °C and 55 °C to mimic the typical physiological operational profile. In principle, once the heat cycle (at 55 °C) had been terminated, the temperature could have been allowed to drift to ambient (22 °C) before repetition. The 30 °C lower limit imposed provides an additional parameter through which to assess the accuracy of the electronic control circuit. In practice, there would be no need to control the “off” temperature within the heater returning to the ambient temperature until the next scheduled release cycle. A comparison between the simulated and actual temperature recorded directly at the heater over five consecutive cycles is detailed in Figure 6B, with no significant deviation between cycles observed. As expected, a deviation from the predicted was observed during both the initial temperature ramp and the subsequent cooling period. It is noteworthy, however, that, the target temperature attained is consistent in each cycle, which confirms the efficacy of the control circuit detailed in Section 2.2. Thermal images of the wax–drug composite before, during, and after heating are shown in Figure 7, highlighting the even distribution of heat across the wax–drug disc. This ensures that the target activation temperature is sufficient to induce transition across the entire surface of the disc.

### 3.2. Release Characteristics

**Figure 8:**
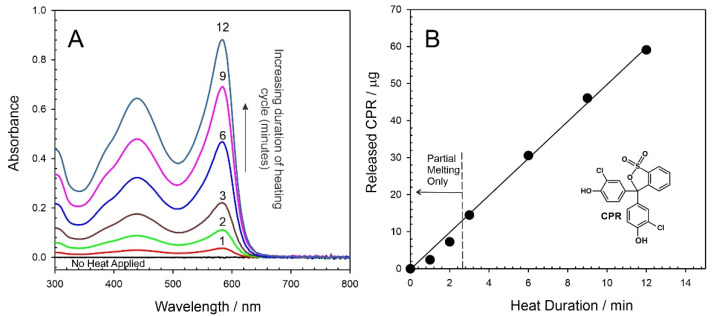
(**A**) UV/Vis spectra detailing the release of chlorophenol red (CPR) from the wax composite into pH 6 buffer as a function of heat duration. (**B**) Estimation of the amount of CPR released during each cycle.

Initial characterisation of the ability of the heater to control the release of a potential drug focused on the use of chlorophenol red (CPR). The cuvette assembly described in Section 2.3 was used throughout and, in this instance, the receiving solution was pH 6 Britton Robinson buffer. Representative UV/Vis spectra detailing the release of CPR as a consequence of differing heat durations (0–12 min) are highlighted in Figure 8A. In the absence of any applied heat, there is no detectable release of CPR. However, upon activating the LIG heater (target temperature of 55 °C), the release of CPR is readily detected, and the spectroscopic profile is confirmed through comparison with CPR standards prepared in pH 6 buffer. Increasing the heat duration increases the amount of CPR released, and a quantitative assessment of the amount (µg per cycle) of CPR released as a function of heat duration is detailed in Figure 8B. Each spectrum represents the first cycle from a new CPR–wax disc in order to avoid any ambiguities associated with memory effects. A linear relationship between heat duration and drug yield is clear for durations between 3 and 12 min. It is noteworthy that there is a slight deviation with a lower-than-expected yield at short durations, which can be attributed to the fact that only partial melting of the wax composite has occurred, and thus, the interface is not fully primed (i.e., the area available for transfer is reduced).

**Figure 9:**
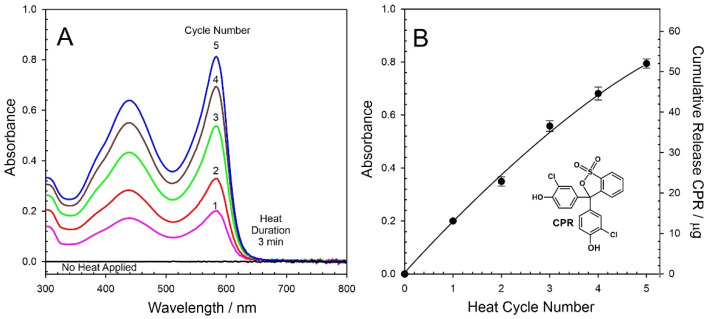
(**A**) UV/Vis spectra detailing the cumulative release of chlorophenol red (CPR) from the wax composite into pH 6 buffer with repetitive heat cycles (heat duration of each cycle: 3 min; cooling period: 10 min). (**B**) Estimation of CPR released.

It can be seen from Figure 8B that the delivery yield can be easily tuned through controlling the heat duration. At 3 min heating duration, some 13 µg of the model drug, corresponding to 1.5% of the total disc capacity, was transferred. Increasing the duration to 12 min similarly increased the yield with 51 µg (5.5%) released. Rather than relying on a single bolus, a critical aim of the proposed system was to enable repeated dosing (ultimately at prescheduled times); therefore, it was necessary to assess the effect of repetitive cycling on drug yield. The heat duration was fixed at 3 min (as per the minimum time required to induce complete phase transition) and UV/Vis spectra detailing the release of CPR into the receiving solution are shown in Figure 9A. In this case, each spectrum represents the cumulative release of CPR into the solution. The process was recorded in triplicate, and a more quantitative measure of the CPR released is highlighted in Figure 9B. It can be seen that the yield per cycle slightly decreases with cycle number, which can be attributed to the gradual depletion of the CPR from the bulk. 

### 3.3. Curcumin Delivery

**Figure 10:**
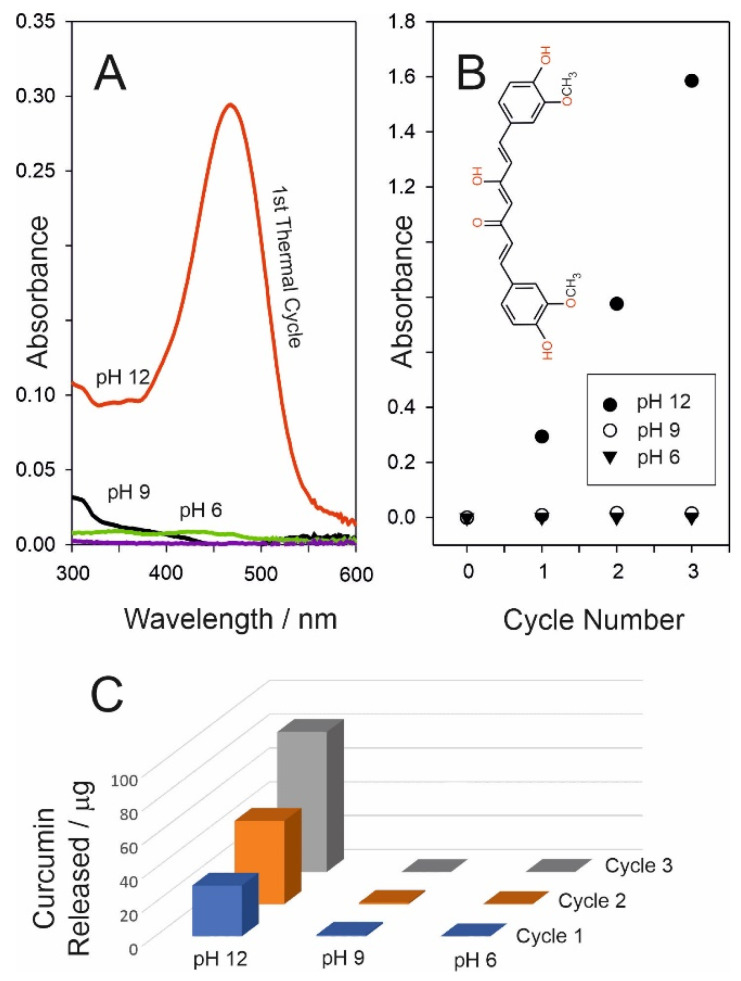
(**A**) UV/vis spectra comparing the influence of pH on the release of curcumin from a wax–curcumin composite film upon heating (3 min). (**B**) Influence of repetitive cycling on the release into buffers of varying pH. (**C**) Comparison of the respective amounts of curcumin released per cycle into the different buffer solutions.

It was originally anticipated that the release dynamics for curcumin would mirror those of CPR, requiring only the simple substitution of one drug component for another. This turned out to be far from simple and failed to take into account the differing natures of the two compounds. While CPR is readily soluble in aqueous solution, curcumin is much more lipophilic and reluctant to partition into pH 6 buffer. The curcumin was effectively retained within the wax irrespective of heat duration. This is highlighted in Figure 10A, where the resulting UV/Vis spectra demonstrated that there is essentially no detectable curcumin within the pH 6 receiving solution after the first cycle (3 min duration). Curcumin (inset Figure 10B) has three hydroxyl protons (OH) with pKa values of 7.8, 8.5, and 9.0 [31]; thus, it is expected to become more soluble in alkaline conditions. It was anticipated that increasing the pH of the receiving solution would, therefore, improve the partitioning of the drug into the aqueous phase. This was observed in practice, but it was only when strong alkaline (0.1 M NaOH) was employed that any significant transfer occurred. In this case, a strong absorption peak (in contrast to pH 6 and pH 9) was observed (Figure 10A), with transfer evident on the first, second, and third heat cycles (Figure 10C), thus confirming successful release. The relative failure to transfer within the pH 9 buffer would indicate that all three hydroxyls must be deprotonated in order to induce transfer.

It is clear that the wax PCM system is far from generic in terms of delivery where facile transfer of nonpolar/lipophilic agents to an aqueous environment is liable to be problematic. It must, however, be recognised that this is an artificial scenario when considering direct transdermal contact and where lipophilic interactions with the skin would dominate. Nevertheless, transfer to an aqueous medium is still relevant when considering controlled delivery from the next generation of smart wound dressing. Rather than direct skin contact, release to the wound fluid/exudate is liable to be a key route for transfer of the therapeutic. In the case of curcumin, this poses a major hurdle where the wound fluid pH can vary between 6 and 9 which, as indicated in Figure 10, is unlikely to facilitate the delivery process.

**Figure 11:**
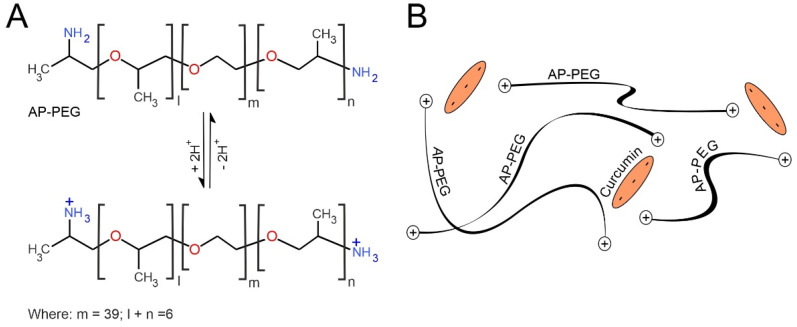
(**A**) Acid–base characteristics of *O*,*O*′-bis(2-aminopropyl) polypropylene glycol–*block*–polyethylene glycol–*block*–polypropylene glycol (AP-PEG) and (**B**) its complexation of curcumin within a polymer network.

An alternative option is to convert the drug to the polar form prior to encapsulation within the wax. This could be readily achieved if an appropriate salt formulation of the drug is available (i.e., diclofenac sodium salt [15]). Unfortunately, there are no commercial salt variants of curcumin. There have been numerous attempts to counter the curcumin solubility issue with complexation, with cyclodextrin [21] and cationic chitosan [22,23] being among the more common [26]. A novel approach was, therefore, taken in which a second phase-change material was added to the drug–wax composite: *O*,*O*′-bis(2-aminopropyl) polypropylene glycol–*block*–polyethylene glycol–*block*–polypropylene glycol (AP-PEG). The latter is basic by virtue of the terminating primary amino groups (Figure 11A) and, therefore, it was anticipated that the direct mixing of molten AP-PEG polymer with curcumin would lead to proton exchange between the amino groups of the former and hydroxyls of the curcumin, essentially forming an “ionic melt”, as indicated in Figure 11B. Ordinarily, the melting point of AP-PEG (22 °C) would be too low for practical use were it to be employed alone but, when the AP-PEG/curcumin mixture is added as a minor component into the wax composite (total curcumin: 1% *w*/*w*), there is a minor depression in the wax transition temperature with the melting point being 38 °C. Ordinarily, this would be too low for practical application and risks accidental activation; however, as mentioned previously, the wax composition can be tuned to a higher melting point through manipulation of the wax composition. As such, the present formulation simply serves as proof of concept rather than eluding to a refined/finalised product.

**Figure 12:**
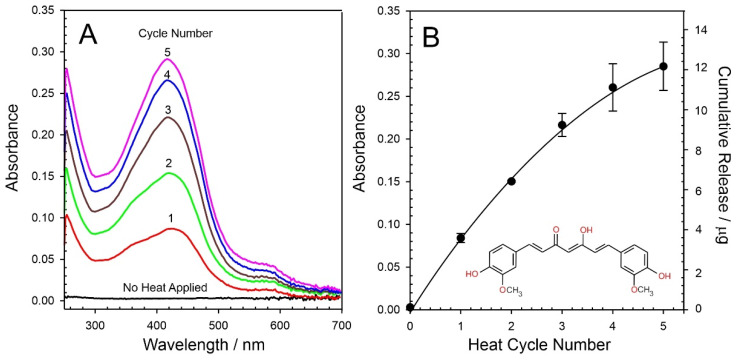
(**A**) UV/Vis spectra detailing the cumulative release of curcumin from a wax/AP-PEG polymer composite film directly into pH 6 buffer. (**B**) Estimation of the amount of curcumin released.

The AP-PEG–curcumin melt is dispersed homogenously throughout the wax as performed previously; however, in this case, the curcumin is predominantly held in the anionic form (Figure 11B). It could be envisaged that, upon reaching the wax interface, the charged nature of the curcumin should enable transfer to the aqueous phase through ion exchange with buffer electrolyte. The delivery of curcumin to a pH 6 receiving solution was again attempted with 3 min heating cycles. The resulting UV/Vis spectra for each cycle are detailed in Figure 12A, where it can be seen that the curcumin absorbance profile increases with each heat cycle. This is in marked contrast to the simple wax–curcumin system where there was almost no detectable transfer (Figure 10A). The absorbance profile was corroborated through comparing the spectra with standard curcumin solution prepared in methanol/buffer (pH 6) mixtures. The AP-PEG polymer has no absorbance in the visible spectrum. A quantitative analysis of the cumulative release of curcumin is detailed in Figure 12B (based on *λ*_max_ = 424 nm, *ε* = 25468 L^−^^1^·mol·cm^−^^1^).

The initial transfer of the curcumin in the first heat cycle (3 min) was found to be 3.5 µg corresponding to only 0.4% of the total curcumin stored within the bulk of the composite disc. This is markedly smaller than the yield obtained for CPR. While the use of the AP-PEG provides a significant improvement in the ability to transfer the curcumin, the poor solubility/hydrophobic nature remains a limiting factor. A comparison between the approach taken here and other systems encapsulating curcumin is provided in Table 1. In most cases, release was measured over a period of 24 h with passive transfer into an appropriate buffer. It is noteworthy that, in the absence of any heat, there is effectively no detectable transfer of the curcumin from the wax–AP-PEG film after 24 h, which confirms that the model drug is securely entrapped.

Activation of the latter releases a bolus corresponding to 0.4% of the total, which can, at first, appear to be inconsequential in relation to the other delivery forms highlighted in Table 1. However, it should be noted that the 3.5 µg released occurs over an activation period of only 3 min. When viewed on these terms and normalising the yields documented in Table 1 to 3 min, the wax–AP-PEG yield is substantially greater that those tabulated. Clearly, there needs to be some caution in performing such comparisons, but the small yield is not as trivial as first inspection would suggest. The true significance, however, relates to the ability to electronically switch the dosing on and, critically, off.

## 4. Conclusions

The model system presented was shown to provide a simple means of encapsulating potential drugs with electronic control over the physical state of the entrapment medium, enabling repetitive scheduled dosing. The blending of model drugs with a hydrocarbon wax mixture is procedurally simple and allows the quick production of thin films exhibiting a solid–liquid phase transition of 43 °C. The graphene-based heater and associated electronics were found to be robust with repetitive cycling between 30 °C and 55 °C facilitating the controlled phase transition of the hydrocarbon wax–drug matrix and therein the release of the drug at the solution–wax interface. Issues of poor partitioning of lipophilic drugs from the molten wax to aqueous buffer were highlighted through the use curcumin as a model therapeutic agent. Modification of the initial wax blend to include an amino-functionalised polypropylene–polyethylene glycol-based polymer to enable the entrapment of the curcumin as the anionic form, however, was shown to greatly improve the release dynamics. This could prove to be a versatile option for when considering drugs with low water solubility.

There are extensive efforts to develop controlled release strategies, and many have gone beyond simple passive delivery. Stimulus-responsive release offers a much more tailored approach to drug delivery but there still remains a requirement for much more control beyond simple initiation. The LIG-based heater system described here is inherently scalable with the added advantages of additive manufacture, allowing translation to microdevices. Moreover, the approach taken here demonstrates how the release process can be electronically tuned—both on and off—to enable repetitive dosing, thus opening up possibilities for scheduled/autonomous treatment regimens. It could also be harnessed as a component within closed-loop smart systems to provide much more regulated dosing and could counter the many issues posed by poor adherence.

## Figures and Tables

**Table 1:** Material strategies and their corresponding release characteristics for the controlled delivery of curcumin.

Matrix/Polymer	Material Type	Release 24 h/%	Curcumin Released	Ref.
Complexation with 2-hydroxypropyl--cyclodextrin in sacran hydrogel film	Film	50	110 µg/cm^2^	[21]
Curcumin nano formulation loaded methoxy poly(ethylene glycol)–*graft*–chitosan composite film	Film	8.4	74 µg/cm^2^	[22]
*N*,*O*-carboxymethyl chitosan and oxidised alginate	Hydrogel	11.8	295 µg	[23]
Tetramethyl orthosilicate (TMOS)	Nanoparticles	81.5	8.15 µg/mg np	[24]
Carrageenan/alginate/poloxamer/curcumin hydrogel film	Hydrogel	88	623 µg/cm^2^	[25]
Paraffin wax–AP-PEG composite	Film	n/a	4.5 µg/cm^2^	This work
